# Bilio-thorax: an unrecognized complication of liver surgery

**DOI:** 10.1016/j.ijscr.2020.04.080

**Published:** 2020-05-14

**Authors:** Asad Ali Kerawala, Abid Jamal

**Affiliations:** The Cancer Foundation Hospital, Karachi, Pakistan

**Keywords:** Biliothorax, Pleural effusion, Liver resection, Oncology, Surgery, Tube thoracostomy

## Abstract

**Introduction:**

Although a rare complication, Biliothorax, or chole-thorax as it has been called in literature, is a devastating entity if not recognized early. Various causes have been reported for biliothorax like inflammatory, abdominal trauma, neoplastic, the most frequent being percutaneous hepatic intervention.

**Case:**

We present the case of a 60 year old male, treated for colon cancer, who developed this complication after liver surgery. He was promptly diagnosed, managed conservatively and responded to our treatment. This has been infrequently reported.

**Discussion:**

Although a rare complication, Biliothorax, is a fatal complication if missed. The presence of bile in the pleural cavity is damaging and can lead to empyema, entrapped lung and even ARDS. Hence it is imperative to recognize early, find the cause and treat appropriately.

**Conclusion:**

Biliothorax is an under reported complication of liver surgery which can have disastrous effects on the patient leading to morbidity and even mortality. It is vital to recognize it early and treat accordingly.

## Highlights

• Metastasis surgery is not very common in developing countries.
• Hence Surgeons are not aware of the complications
• Bile presence in Pleural cavity is rare and detrimental to patient.
• High index of suspicion is required in such cases.

## 1 Introduction

Fistulas between the biliary tree and the chest have been a reality after liver surgery. Most of them present as pleural effusions, however, cases of bronchial fistula are also not unheard of. This complication as a result of any liver intervention is devastating and if not recognized early can lead to dire consequences for the patient. The patient may develop respiratory complications like pneumonia, Empyema, ARDS all of them fatal. Presence of bile in the pleural cavity has been infrequently reported in the literature even though it occurs quite often. Physicians who have a high index of suspicion usually diagnose this before any harm is done but at times it is diagnosed too late and the patient suffers. We report one such case of Bilio-pleural fistula in a cancer patient. The work has been reported in accordance with SCARE criteria [1].

## 2 Case report

A 60-year-old man presented to our clinic for follow-up and reversal of his stoma. He underwent a right extended hemicolectomy with diversion loop ileostomy 1 year back for adenocarcinoma of hepatic flexure and received 4 cycles of FOLFOX (5-Fluorouracil, Leucovorin and Oxaliplatin) subsequently. He stayed fine for a year and then started having rising levels of CEA (carcinoembryonic antigen). His CT and PET scan showed high metabolic uptake in segment VIII of liver. No evidence of local recurrence was found. The case was discussed in tumor board at our institution and it was decided to perform a metastatectomy of his liver lesion along with the reversal of his stoma (Picture 1, Picture 2).

**Picture 1:**
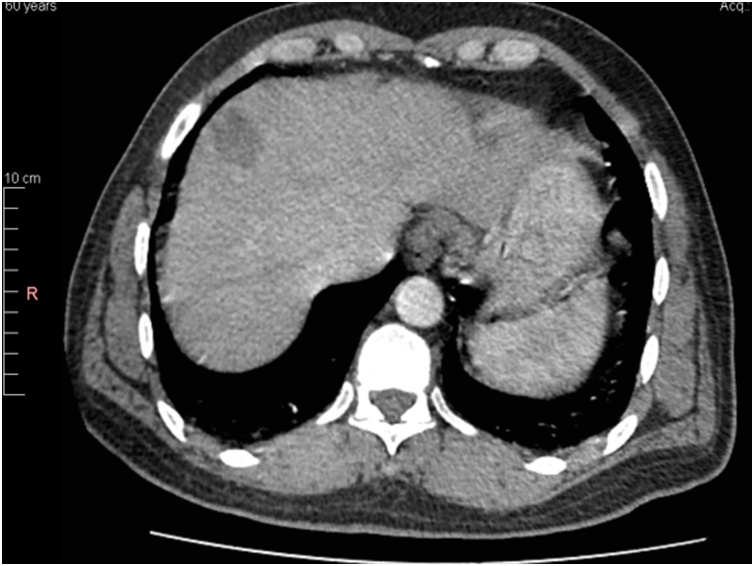
CT scan showing single liver lesion.

**Picture 2:**
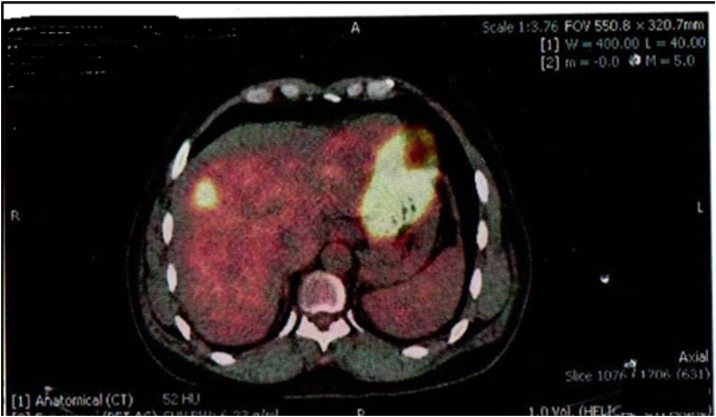
PET scan.

Per operatively many adhesions were found surrounding the liver and it was a technically difficult resection. Intra operative ultrasound was carried out to localize the lesion and its proximity to major vessels. No other lesions were found. A 7 cm by 5 cm wedge of liver segment VIII was resected with Aquamentis/CUSA. Stoma was reversed and the abdomen was closed. No intraabdominal drains were placed. Post operatively patient stayed fine and was discharged home on 12th Post-operative day after he was orally tolerating and fully mobilized. On discharge no intra-abdominal collection was found on ultrasound.

7 days later the patient presented in emergency department with severe dyspnea and fever for 1 day. His saturation on room air was 84% and respiratory rate was 34.

On examination there were decreased breath sounds on right side of chest with dull percussion note. A Chest Xray revealed massive pleural effusion and tracheal deviation to the left side. Immediate tube thoracostomy was done and 2600 purulent fluid was drained. The patient got better. The next day chest tube drained golden colored fluid. The fluid was sent for bilirubin levels and high levels were found confirming our suspicion of biliothorax. Total leucocyte count was 17,000 and total bilirubin was 4.2. He was started on broad spectrum penicillin – Tazobactam with Piperacillin.

The patient kept draining 300 mL, 250 mL of bile per day for 7 days. No abdominal collection was found. An ERCP and sphincterotomy was planned but the sphincter was in spasm and a sphincterotomy could not be performed fully. He was planned for another attempt the next day but post ERCP, the drainage started reducing to 150 mL, and then 100 mL and the patient was discharged home with the tube.

The drainage eventually stopped and the chest tube was removed on 23rd day. The reason was probably the fact that the bile leak collected and because of no presence of drain in the abdomen, it made its way in the Pleural cavity through the pleuro – peritoneal canal. Another cause could be a diaphragmatic injury which was not identified per operatively, but there was no pneumothorax to suggest that (Picture 3).

**Picture 3:**
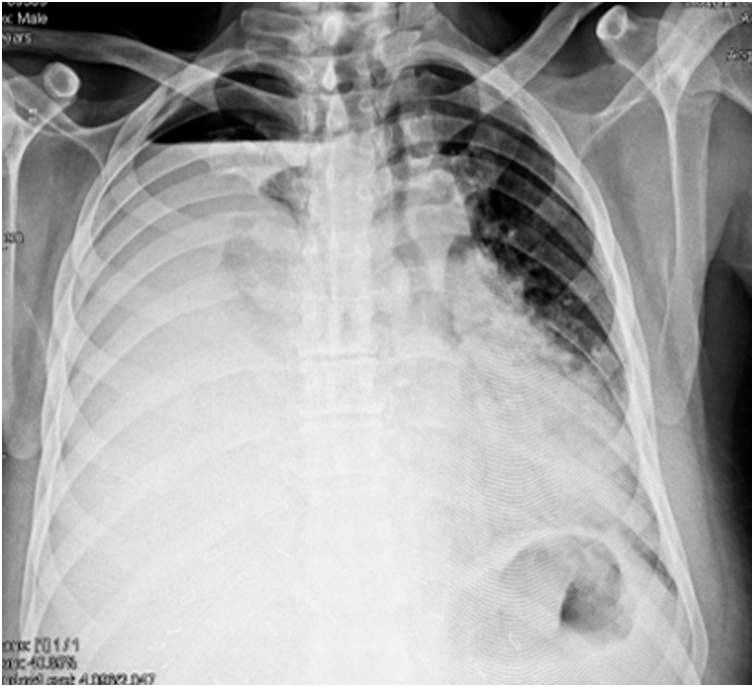
Right sided Pleural Effusion.

## 3 Discussion

Biliothorax was first reported by Graham when he presented two patients in 1897 [2]. He called them Broncho biliary fistulas. Although a rare complication, Biliothorax, or chole-thorax as it has been called in literature, is a devastating entity if not recognized early. The presence of bile in the pleural cavity is damaging [3,4] and can lead to empyema, entrapped lung and even ARDS. Hence it is imperative to recognize early and treat appropriately. Various causes have been reported for biliothorax like inflammatory, abdominal trauma, neoplastic, the most frequent being percutaneous hepatic intervention [[5], [6], [7]]. In percutaneous biliary intervention various theories have been proposed for the cause of biliary pleural fistula. The catheter tract may be traversing through the pleural causing an iatrogenic fistula. Secondly due to obstruction in the biliary tract, the high pressure in the system may cause bile to drain through the pleura7. Besides this, the negative pressure during inspiration may suck the bile from the high-pressure biliary tree to the low-pressure pleural cavity. Hepato biliary surgery associated with biliopleural fistulas have been reported infrequently. A defect in the diaphragm causes any bile leak to reach the pleural cavity. Over time the fistula matures unless drained. In our case we were still not sure about the cause.

Diagnosis of biliothorax requires a high index of suspicion in any patient undergoing hepatic surgery. A ratio of pleural bilirubin to serum bilirubin > 1 is diagnostic. Imaging studies like CT may show the fistula after a few days after the tract has matured. On ERCP, contrast spillage through the biliary tree into the pleura is noted and is confirmatory of the diagnosis.

There is no consensus on the treatment of this rare entity. Although most case reports advocate a conservative approach with appropriate drainage through chest tube [8,9]. It can be avoided by per operative check for bile leaks using propofol or methylene blue but the data for this seems to be scarce. Intra-abdominal drains may also prevent it, by draining the bile externally instead of towards the pleura. We employed a non-operative approach for this patient starting with tube thoracostomy and then endoscopic sphincterotomy. Since there was no abdominal collection no drainage was required there. Other reports also show successful treatment of the disease with conservative measures [10,11], sparing the patient another major surgery and its associated morbidity.

## 4 Conclusion

Biliothorax is an under reported complication of liver surgery which can have disastrous effects on the patient leading to morbidity and even mortality. It is imperative to recognize it early and treat accordingly.

## Declaration of Competing Interest

There is no conflict of interest or financial disclosures.

## Funding

No funding was received.

## Ethical approval

Case report publication does not require an ethical approval at our institution, however informed written consent was taken from the patient and his family. It was made sure that his identity will be kept a secret at all levels.

## Consent

Written informed consent was taken from the patient and his family regarding publication of this case report and accompanying images. It was made sure that his identity is kept covert at all levels. A copy of written request is available for review if requested.

## Author contribution

Kerawala, Asad Ali : Drafting of article, Data collection, Study design and concept.

Jamal, Abid: Study conception and design, Drafting of manuscript, Critical revision.

## Registration of research studies

NA.

## Guarantor

Asad Ali Kerawala.

Email: asadali4@yahoo.com

## Patient’s consent

Informed consent was taken from the patient about this case report.

## Provenance and peer review

Not commissioned, externally peer-reviewed.

## References

[1] Agha R.A., Borrelli M.R., Farwana R., Koshy K., Fowler A., Orgill D.P., For the SCARE Group (2018). The SCARE 2018 statement: updating consensus surgical CAse REport (SCARE) guidelines. Int. J. Surg..

[2] Graham J.E. (1897). Broncho-biliary fistula. Br. Med. J..

[3] Porembka D.T., Kier A., Sehlhorst S., Boyce S., Orlowski J.P., Davis K. (1993). The pathophysiologic changes following bile aspiration in a porcine lung model. Chest.

[4] Perez M.J., Briz O. (2009). Bile-acid-induced cell injury and protection. World J. Gastroenterol..

[5] Yu E.Y., Yang F.S., Chiu Y.J., Tsai F.J., Lu C.C., Yang J.S. (2018). Late onset of biliopleural fistula following percutaneous transhepatic biliary drainage: a case report. BioMedicine.

[6] Strange C., Allen M.L., Freedland P.N., Cunningham J., Sahn S.A. (1988). Biliopleural fistula as a complication of percutaneous biliary drainage: experimental evidence for pleural inflammation. Am. Rev. Respir. Dis..

[7] Celis C.A., Tobon M., Ramirez R., Garcia M., Suarez J. (2015). Cholethorax secondary to percutaneous transhepatic gallbladder drainage: a case report. InB39. Do you want to know a secret?. Case Rep. Pleural Dis..

[8] Nikumbh T.N., Barot P.V., Kurlekar U.A., Bapaye A.M. (2018). Endoscopic management of bilio-pleural fistula following thoracoabdominal trauma. Oncol. Gastroenterol. Hepatol. Rep..

[9] Petri C.R., Majid A., Anandaiah A. (2019). A man with biliary Sepsis and an enlarging pleural effusion. Ann. Am. Thorac. Soc..

[10] Pew K., Thomas S. (2019). Bilious airways: a case of bilothorax following liver transplantation. InB43. Pleural Dis. Case Rep. I.

[11] Shah K., Ravikumar N., Uddin Q.K., McGee W., Farmer M.J. (2019). Bilateral bilothorax: an unusual cause of bilateral exudative pleural effusion. Cureus.

